# The Naked Neck Gene in the Domestic Chicken: A Genetic Strategy to Mitigate the Impact of Heat Stress in Poultry Production—A Review

**DOI:** 10.3390/ani13061007

**Published:** 2023-03-10

**Authors:** Elisabete Fernandes, Anabela Raymundo, Luisa Louro Martins, Madalena Lordelo, André M. de Almeida

**Affiliations:** LEAF—Linking Landscape, Environment, Agriculture and Food Research Center, Associated Laboratory TERRA, Instituto Superior de Agronomia, Universidade de Lisboa, Tapada da Ajuda, 1349-017 Lisboa, Portugal

**Keywords:** heat stress, poultry, genetics, naked neck

## Abstract

**Simple Summary:**

Temperature and humidity are environmental factors with significant impact on animals. This challenge is not exclusive to tropical countries, as climate change has a worldwide impact. Birds are particularly sensitive to environmental changes, and high temperature causes significant financial losses. Several strategies can be used to mitigate the impact of heat stress in poultry. The use of strains tolerant to high temperature may be an interesting solution. In this review, we address the major effects of heat stress in poultry and how the naked neck gene can be used to mitigate such adverse effects.

**Abstract:**

The poultry sector is one of the most important food industries in the world. Poultry production generates high-value protein products (meat and eggs) that are produced efficiently without the need for large areas. In poultry production, especially in the tropics, environmental factors, such as temperature and humidity, play a major role. Heat stress (HS) causes behavioral, physical, and physiological changes in poultry, with severe financial impacts. Therefore, it is important to find strategies to minimize it. The naked neck (Na) is an autosomal, incompletely dominant gene. Compared with normal feathered birds, these animals are known for their ability to adapt, perform, and reproduce under hot and humid climate conditions. Due to the absence of feathers on the neck, these animals increase heat dissipation, alleviating adverse heat effects, especially on productive performance. Genetic improvement of heat tolerance may provide a low-cost solution, of particular interest for developing countries in the tropics. The focus of this review is to evaluate the impact of HS in poultry with a special emphasis on the advantages of using the Na gene.

## 1. Introduction

Consumers are increasingly concerned about what they eat and what consequences their choices have on the environment. Therefore, they are currently searching for more sustainable alternatives with additional health benefits. The poultry industry is considered the most efficient animal protein production system (meat and eggs). Poultry meat production is considered to have the least impact on climate change, contributing 9.8% per kg of meat, 0.3 and 27.2 percentage points less when compared to beef and pork, respectively [1]. Poultry meat contains a low amount of saturated fatty acids, and both meat and eggs are remarkable sources of protein, fat, and micronutrients, which play an important role in human nutrition [2], especially in the tropics, where it becomes a cheaper source of protein when compared to other livestock species [3]. In 2019, the FAO (Food and Agriculture Organization) [4] estimated that 131.6 million tons of annual global chicken meat production contributed to roughly 39% of the total global meat production.

High year-round temperatures, intense sunlight, and two different seasons (rainy and dry) characterize tropical climates. Poultry production in the tropics is generally associated with free-range production systems, meaning that environmental issues are among the biggest challenges [5]. Climate change is now a reality and a challenge in animal production. Unlike ruminants, most monogastric animal production is carried out indoors, so these animals would be less susceptible to climate change. However, due to growing concerns about the consumption of food from less intensive systems, the increase in energy costs (for example, with ventilation), and animal welfare, free-range production systems for monogastric animals are now common in industrialized countries [6].

All species respond to environmental challenges; however, due to the absence of sweat glands, birds are more sensitive to high temperatures. High temperatures negatively influence performance (e.g., growth rate, feed intake and utilization, body weight, egg production and quality, meat quality) as well as physiological and immunological responses of the birds, causing unfavorable consequences and, in extreme situations, leading to death, representing substantial economic losses [7].

The main goal in intensive poultry production systems is to produce the highest amount of meat possible in the shortest time and at the lowest cost, creating an affordable source of protein. To achieve this, over the last decades, genotypes with higher growth rates were selected. However, a faster metabolism leads to higher metabolic activity and higher heat production. Therefore, poultry genotypes became increasingly sensitive to high temperatures, requiring a lower ambient temperature to reach their maximum growth potential [8]. The thermoneutral zone of chickens depends on several factors. These include, for instance, the amount, shape, and distribution of feathers. At low temperatures, feathers have important insulation properties; however, at high temperatures, they prevent heat dissipation [9].

The Na gene reduces feather coverage (relative to body weight) in chickens by about 20% in heterozygous (Nana) and 40% in homozygous (NaNa) when compared to normally feathered animals (nana) [10]. Since these animals have less plumage, they can better tolerate high temperatures and may represent a low-cost solution that is particularly attractive to developing countries with warm climates. Additionally, in tropical countries, there are frequent infrastructural problems and a scarcity of plucking equipment and techniques. Therefore, the Na gene may represent another economic advantage. Indeed, due to their reduced feather coverage, these animals have a lower operating cost, as they make plucking easier and faster. 

Several reviews on HS and the effect on the poultry industry may be found in the literature. This review aims to present a different point of view focused on the use of selected animals as a solution tolerant to high environmental temperatures. As such, for contextualization purposes, we first summarize the general effects of HS on poultry production and then present the potential strategy of using the Na gene to mitigate such effects.

## 2. Impact of High Ambient Temperature in Poultry

### 2.1. Heat Stress

Stress can be defined as any biological response when an animal receives a stimulus/threat that alters its homeostasis or physiological balance [11]. As it has long been described by Selye [12], there are two important concepts: stress, “the nonspecific response of the body to any demand”, and stressor, “an agent that produces stress at any time”. HS can affect all poultry breeds at any age and results when the animal struggles to dissipate the heat produced internally, causing a negative balance [13]. In adult birds, body temperature ranges between 41 and 42 °C [14]. To achieve the animals’ full potential, it is important that adult animals are in their thermoneutral zone: 19–22 °C for laying hens and 18–22 °C for broilers [15]. An increase in such temperatures may cause HS. In addition to temperature, it is very important to monitor relative humidity. The combination of temperature and humidity, known as the temperature–humidity index (THI), measures the degree of discomfort during high temperatures [16]. Thus, stress can be classified according to the combination of these two factors and the duration of exposure. If we combine high temperatures with high humidity for a short and sudden period, it will result in acute HS. On the other hand, if the time is extended, it will result in chronic HS. Acute stress can lead to high mortality rates due to suffocation, and chronic HS can significantly affect growth performance [17].

### 2.2. Mechanisms of Body Heat Regulation

**Figure 1:**
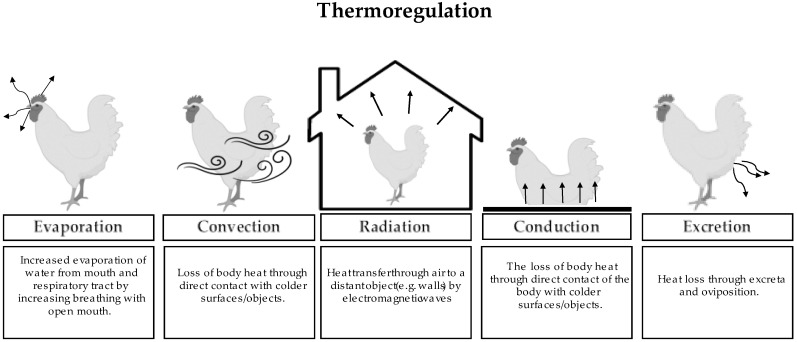
Definition and illustration of the thermoregulation mechanisms five (evaporation, convection, radiation, conduction, and excretion) of body heat in poultry.

Presently, due to extensive genetic selection and nutritional strategies, laying hens can produce around 250 eggs in the first year [18] and broilers are ready for market in 35–42 days, with 2.5–3.0 kg of body weight [19]. However, as mentioned, faster metabolism leads to higher metabolic activity and therefore body heat production. Thus, fast-growing or highly productive genetics will be more sensitive to high environmental temperatures [20]. Birds are homeothermic animals, as they do not depend on environmental temperature to maintain body temperature [14]. There are five mechanisms (Figure 1) involved in the regulation of body heat. Convection, the most effective way to reduce HS, is heat loss through the passage of air over the animal. However, this solution may require the use of ventilation systems. Radiation is the energy that propagates through an electromagnetic wave to surrounding surfaces. It only occurs if the internal temperature of the animal is higher than the temperature of the surrounding environment. Conduction is the loss of heat through direct contact with surfaces that are cooler. The latter is, however, normally irrelevant, because heat loss is insignificant [21]. These three mechanisms only work if the ambient temperature is below or within the thermoneutral zone. On the other hand, when we have high temperatures, the evaporation of water from the mouth and respiratory tract will depend on panting to release body heat. However, to be effective, it is important that the humidity of the air is not high [22]. Excretion is heat loss through the excretion process, increasing water consumption and producing wet excreta [21].

### 2.3. Physiological Effects of Heat Stress

To decrease body temperature and thus maintain homeostasis at high temperatures, birds undergo a vast array of physiological responses. Such responses, which depend on their intensity and duration, affecting, in turn, different physiological functions, may occur at any stage of the animal’s life. Physiological behavior has, in turn, an influence on animal performance [23,24]. Since chickens do not have sweat glands, they are very sensitive to HS. In addition to aiding in respiration and flight, poultry air sacs are involved in regulating body temperature. Air sacs are thus fundamental in gas exchange since they act in the movement of air by pressure difference. However, increased panting exhales more carbon dioxide and induces a higher blood pH (respiratory alkalosis) [25]. This effect is particularly important in laying hens as it affects the availability of circulating free bicarbonate and calcium for eggshell mineralization, thus reducing eggshell strength [26]. For each unit increase in THI, there is an increase of 0.56 breaths per minute [27]. According to a recent study conducted by Abioja et al. [28], at 31 °C and 69% humidity (THI 84), the respiratory rate can be 40–43 BPM and the heart rate 320–350 bpm. A heart rate over 300 bpm is an indicator that the birds are under stress. In the same study, respiratory rate was different between sexes, suggesting that females are more affected than males.

**Figure 2:**
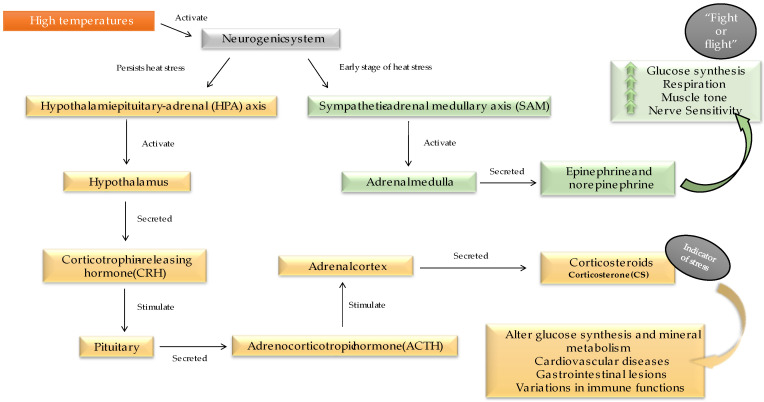
Physiological response triggered in the early stage of heat stress (green part) and persistent heat stress (yellow part) and what implications can be caused by each when poultry are subjected to high temperatures.

When an animal faces a stressor, the neurogenic system is activated. Indeed, in the early stages of HS, the sympathetic–adrenal medullary axis (SAM) is activated and regulates homeostasis. However, when stress persists for an extended period, the hypothalamic–pituitary–adrenal (HPA) axis is activated [29]. The mechanisms and effects of the physiological stress response in poultry are shown in Figure 2.

The thyroid hormones thyroxine (T4) and 3,3′,5-triiodothyronine (T3) regulate body temperature and metabolic activity and play an important role in development and growth [30]. Several studies demonstrate that the concentration of T3 decreases with increasing temperature [7,11,31,32]; however, contradictory results are found for T4 [33,34,35]. In addition, the thyroid gland is directly involved in sexual development and the reproductive function of animals, so it is expected that changes, such as HS, would influence reproductive performance [36].

### 2.4. Effect of Heat Stress on Productive Performance and Behavior

**Figure 3:**
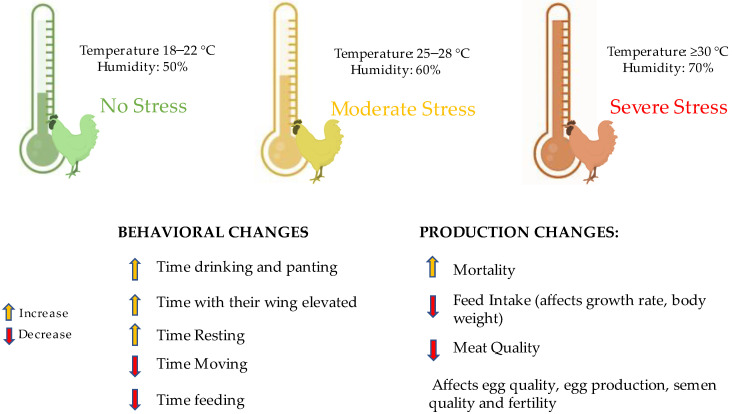
Stress type (none, moderate, and severe) implications and the combined effect of different temperatures and humidity on birds and their implications on performance and behavior.

As mentioned, for birds to reach their maximum productive potential, it is essential for adult animals to be in their thermoneutral zone. When exposed to high temperatures, birds try to dissipate excess heat through specific behaviors. These include reduced feed consumption, spreading their wings, spending more time lying down, and panting [13]. Another behavioral change with physiological implications is a 5–10% increase in water consumption, as they dissipate heat through wet excreta [20]. Such behavioral changes influence the animal’s performance (Figure 3). 

A previous study comparing the thermoneutral state (27 ± 2 °C) and high-temperature conditions (37 ± 2 °C) showed a reduction in daily feed intake and average daily gain of 31.9 and 15.8%, respectively [37]. This study is in accordance with the results found by Awad et al. [38] who conducted a study using Cobb 500 and Ross 308 broilers at 34 °C in a 13-day trial and found a reduction in feed intake and body weight gain of 8–9% and 17%, respectively, and an increase in feed conversion ratio of 9–10%. 

Laying hens are also strongly affected by high temperatures, which lead to poor egg quality and shell thickness and a decrease in production. Additionally, when feed intake decreased, calcium intake also decreased, causing imbalances in calcium levels and plasma protein [13]. 

Therefore, HS influences the bird’s physiology, which in turn affects the productive performance of both broilers and laying hens, ultimately contributing to a large financial impact.

### 2.5. Effects of Heat Stress on the Immune Response

Birds use energy from feed for growth, reproduction, and immune system development. A strong immune system is the key to better production performance; thus, it is related to the animal’s health. Therefore, the efficient conversion of feed into its basic components for optimal nutrient absorption is vital. Gut health plays a key role, as it combines physiology, immunology, nutrition, and the microbiome. The gut acts as a barrier that eliminates toxins and infectious agents. When gut health is compromised, digestion and nutrient absorption are affected, which in turn can have a detrimental effect on feed conversion and increased susceptibility to diseases, and in extreme cases, it may lead to death [39,40]. Among other factors, the gut microbiome is influenced by temperature because several types of bacterial pathogens may inhabit the gut and thus disturb its ecosystem [41].

During HS, the physiological changes to maintain body temperature reduce immune response and increase the likelihood of animals contracting diseases. The central nervous system (CNS) modulates the immune response through a bi-directional complex network between immune, endocrine, and nervous systems. The immune response can be altered mainly through pathways of the sympathetic–adrenal medullary (SAM) and the hypothalamic–pituitary–adrenal (HPA) axes [13]. Under HS conditions, laying hens exhibited lower liver weights and lower relative weights of the thymus and spleen [42]. In broilers, it has also been reported that there is a reduction in lymphoid organ weights [43]. Another negative effect is the increase in pathogenic bacteria, such as *Salmonella* sp., *Clostridium* sp., and *Escherichia coli*, because of increased intestinal permeability due to HS [44]. Although several studies have been conducted to understand how stress can affect the immune response, there is still not a full understanding of the immune response to HS in poultry with reference to genetic and cellular mechanisms.

## 3. The Naked Neck Gene (Na) as a Strategy to Mitigate Heat Stress

Temperature is the environmental factor with the greatest impact on poultry production. Such impact can be further aggravated if accompanied by high relative humidity and affects both broilers and laying hens. It is a problem that can involve many financial losses. Several studies have been conducted using different strategies to mitigate such impacts. These strategies can include feeding strategies, environmental modifications, or genetic selection. As far as feeding strategies are concerned, we can find solutions that are easier to implement, such as formulating diets according to the metabolism of the bird, wet feeding, feed restrictions, or some more expensive solutions, such as adding additives to the feed. Environmental strategies can also be quite expensive, namely, refrigeration, which furthermore has environmental implications in the form of greenhouse gas emissions. On the other hand, the selection of genetics better adapted to a certain climate is perhaps the most feasible strategy. There are several genes, namely the Na and the frizzle (F), that present a more economical, sustainable, and attractive answer to such challenges [45].

### 3.1. Origins and Physiological Traits of Naked Neck Animals

The origin of the Na gene in chickens is not fully known. It is believed that it originated in Asia and was later developed in Germany. Due to the physical similarities to the domestic turkey, these animals were believed to be hybrids and were thus nicknamed Türken (Turkish in German). Today they are found all over the world, being quite common in Europe and South America [46].

**Figure 4:**
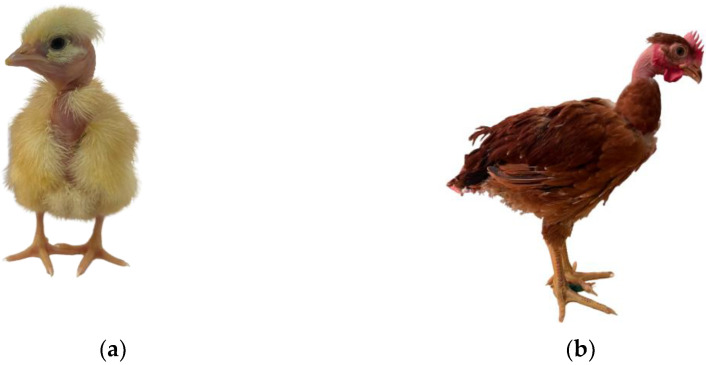
Naked neck birds. (**a**) Young heterozygous naked neck chick with a small tuft of fathers on the neck. (**b**) Adult heterozygous naked neck animal with a small tuft of fathers on the neck.

The Na gene, with the symbol assigned by Hertwig [47], is an autosomal, incompletely dominant gene. Initially the Na gene was identified as belonging to intergenic DNA of chromosome 1 [48]; however, it was recently associated as belonging to a region on chromosome 3. Being insertion unique to naked neck animals, it was not present in other animals. It is at the embryonic stage, namely at 7 days of incubation, that the number and distribution of the adult bird’s feathers is defined. The expression of GDF7—Growth Differentiation Factor 7 gene (also known as BMP12), expressed in developing bird skin—was found to be higher in naked neck embryos. So, it was concluded that the Na phenotype is caused by an increased expression of GDF7. GDF7 suppresses the development and distribution of feathers by the sensitizing action of retinoic acid, derived from vitamin A, mostly in the vent and neck region [49]. The body responds to the action of GDF7 differently, with the neck region being a particularity sensitive zone. The heterozygous animals (Nana) are different and easily distinguished from homozygous animals (NaNa), as they exhibit different phenotypes. NaNa birds have no plumage in the neck region, while Nana animals have a small tuft of feathers [48], as shown in Figure 4. Compared to the normal plumage (nana), the reduction in feathers is about 20% in heterozygous and 40% in homozygous animals. Despite the difference in the percentage of plumage, it is not always easy to distinguish them. This is related to the incomplete dominance expression of the Na gene. Incomplete dominance delays the fixation of one allele and reduces heterosis when compared to complete dominance [50]. The reduction in plumage gives these animals the ability to better dissipate heat when exposed to high ambient temperatures [10]. In broilers, the weight of the feathers at slaughter is about 5% of the animal’s weight, most of which is protein. Since not so much protein is needed for feather development, there is an extra amount that will be channeled, for example, into the growth of the animals [51].

When animals are subjected to high temperatures, a rapid response of heat shock proteins (HSPs) occurs. The function of these proteins is to protect organs and cells from the negative effects of HS [52]. Among the various HSPs produced, one that shows the highest correlation with thermal tolerance is HSP70 [53]. The greater the amount of HSP70 produced, the greater the animal’s tolerance to HS [54,55]. The Na genotype has a higher expression of the HSP70 gene when compared to animals with normal plumage [56]. For those reasons, when the Na gene is present, the animals can more easily adapt to high temperatures, with lower mortalities and higher performances when compared with the normally feathered birds [57].

### 3.2. Effects of the Naked Neck Gene on Poultry Growth

Over the years, several studies [58,59,60,61,62] have shown that, at high temperatures, the Na gene had a favorable effect on growth performance. Since they have a higher resistance to HS, these animals can mitigate adverse effects, for instance, on body weight gain, feed intake, and feed conversion ratio [62].

Up to 32 °C, weight gain can be improved by 7–38%, feed intake can increase 10–20%, and feed conversion ratio (FCR) can be reduced by 10% [58,60,61,63] when compared to normally feathered animals. The differences depend on the crosses, growing period, and if we are using heterozygous or homozygous animals. A recent study performed on 320 naked neck chickens of four different phenotypes (black, white-black, light brown, and dark brown) concluded that there are significant differences in growth, cholesterol content, and morphometric characteristics, where the light and dark brown phenotypes showed better results [64]. 

On the other hand, at temperatures above 32 °C, the growth of the animals can be compromised, particularly in normally feathered birds, whereas naked neck animals show better growth rates [59,60]. In Table 1, several literature references are summarized on the effect of the Na gene on growth parameters at different temperatures. They all confirm the higher growth performance of NaNa and Nana animals at high temperatures when compared to nana animals. Overall, they demonstrate the higher growth ability of Na animals under high ambient temperatures.

### 3.3. Effects of the Naked Neck Gene on Meat Traits

In poultry meat, taste, texture, appearance, color, and juiciness are the most important physical quality attributes [65,66]. It is important to produce a carcass of good quality and obtain a maximal yield with limited fat content. Additionally, dressing percentage, or carcass yields, is one of the most important traits to evaluate carcass and meat quality. 

In Table 2, we summarize different references on the effect of the Na gene on meat parameters at different temperatures. According to several studies [61,62,67,68], even when animals are not under HS, Nana and NaNa animals have higher percentages of carcass yields and typically have higher adult weights, which, in turn, will translate into a higher meat yield than normally feathered animals [61,66,69]. Increases in live weight in naked neck strains depend on whether they are homozygous or heterozygous. This is a feature of major interest for the producer but also for the consumer [66]. Generally, heterozygous animals show higher live weights [70]. The flavor of meat is affected by its fat content. To facilitate heat dissipation, NaNa and Nana chickens have a reduction in fat in the skin and breast muscles that can influence meat flavor [71]. A relation exists between body fat and heat tolerance [72] that negatively affects meat quality. One theory for this relationship is based on the possibility that these animals use a greater amount of energy for thermoregulation. This explanation agrees with the study conducted by Raju et al. [71], who found a reduction in abdominal fat when animals were subjected to temperatures between 28 and 37 °C (−28.62%), which was not the case when the same strain was subjected to temperatures between 19 and 26 °C. However, contradictory results are also found in the literature [67,70]. On the other hand, there is greater protein and amino acid availability, as they are not used for feather development, but rather channeled to breast and thigh muscle [10]. 

Meat composition, particularly the fatty acid profile, is an extremely relevant factor, with an impact on meat sensory acceptance and health benefits. Generally, there is a higher percentage of n-3 PUFA in the meat of slow-growing broilers when compared to fast-growing animals [73]. According to a study by Duah et al. [74], the meat of the Na genotype had lower fat and cholesterol content (*p* < 0.05). 

### 3.4. Effects of the Naked Neck Gene on Egg Production Traits

In egg production, the most important trait is the egg production rate, or the number of eggs produced/time unit. This is the indicator of production efficiency and is of extreme importance. In addition to this factor, it is also important to monitor egg weight, shell integrity, and internal quality, as these factors are related to the economic value of the egg [75]. 

When addressing egg production and quality, there are several studies that report the advantage of using the Na gene at high or moderate temperatures. At 30 °C, the use of Na genotype may lead to an annual increase of 15 eggs per hen [10]. This study agrees with [76], which observed an increase of 20% in egg production when comparing naked neck (Nana) Criollo hens with normal plumage (nana) animals. Nevertheless, at low temperatures, these animals produce fewer eggs and have an overall lower egg mass [77].

Egg quality is influenced by genetics, environment, and feed intake. Eggshell quality can be improved using Na genotype hens due to the reduction in feathers, as it is easier to absorb solar radiation, which in turn increases vitamin D3 synthesis [78]. Another advantage is the ability of naked neck hens to produce eggs with better shell quality under HS. As mentioned earlier, with increased respiration alkalosis, there is less availability of free bicarbonate and calcium for eggshell mineralization, something that these animals can better control [79].

Haugh Units can be a measure for the internal egg quality. The higher the number, the better the quality of the egg. The use of heterozygous naked neck (Nana) genotypes can significantly increase Haugh Units (+3.2%) when compared to normally feathered animals [80].

### 3.5. Effects of the Naked Neck Gene on Egg Reproductive and Egg Hatchability Traits

Fertility (% of incubated eggs that are fertile) and hatchability (% of fertile eggs that hatch) are the two main parameters for day-old chick production. The presence of lethal genes, nutrition, storage conditions, and genetics are all factors that can influence such traits [81]. Eggs from animals with heat tolerance genes, namely Na, can be more fertile than eggs from animals without them [82]. Sexual maturity in broilers and hens is an important parameter for commercial breeders, since the earlier it is reached, the longer the reproductive period will be. Na chickens have earlier sexual development [9], which is in accordance with Abou-Emera et al. [80], who conducted a study and concluded that Na animals reached sexual maturity earlier (−14 days) than normally feathered hens. In the same study, authors also observed that the naked neck animals obtained a higher mature weight (+150 g), which translated into a higher egg production rate. Despite their early sexual maturity, several studies prove that the Na gene is correlated with some problems in relevant reproductive parameters, such as lower sperm motility, higher counts of abnormal sperm cells, and poor egg hatchability [83]. However, under HS, these animals show higher fertility, hatchability, and number of live chicks when compared to the normal plumage genotype [79,84]. Regarding embryonic mortality, it seems to be more pronounced in homozygous animals. The cause for such an increase in late-stage embryonic mortality is not fully understood, but it is thought to be related to the lethality of the Na gene when present in a dominant form. Therefore, the homozygous (NaNa) genotype is more affected than heterozygous (Nana) animals [84]. Under natural incubation conditions, low hatchability may be related to the fact that these animals have a reduction in feathers, which is important for providing the appropriate brooding temperature [85]. For this reason, genetic selection and crossbreeding with other breeds can be the solution to improve these parameters.

### 3.6. Effects of the Naked Neck Gene on General Animal Health

Outdoor and organic production systems have become increasingly important, with the society’s growing concern on animal welfare and environmental impact, in addition to human nutrition. In such systems, the immune system and the general health of the animals have even greater importance. Currently there are no regulations/guidelines for the mandatory use of certain strains depending on the production system. However, due to their characteristics, there are more and less appropriate genotypes. In outdoor production systems, it is necessary that the animals always be in a good state of health whilst being physiologically and morphologically adapted to outdoor spaces and more resistant to diseases and temperature variations [86]. Fast-growing strains are more likely to develop leg problems, thereby restricting mobility and welfare, since there is a direct relationship between the growth rate of broilers and bone health [87]. Some studies show that there is a higher resistance to bacterial, viral, and parasitic infections in animals of the Na genotype when compared to normally feathered birds [61,76,88]. A recent study concluded that Na strains have the most uniform microbiological diversity. Larger populations of beneficial microbes (*Lysinibacillus* and *Brevibacillus*) were found in comparison to potentially pathogenic bacteria (*Clostridium*), suggesting that these animals have higher gut health [89]. However, this is a recent area, and more studies should be conducted, as contradictory results have also been found [74].

## 4. General Considerations

**Figure 5:**
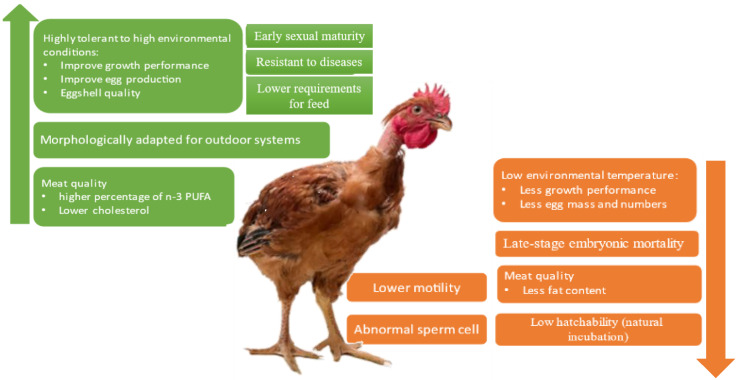
Summary of the advantages (green) and disadvantages (orange) of the naked neck gene in the different parameters and their possible productive implications.

The advantages and disadvantages of the Na gene in the different parameters are summarized in Figure 5. Some of them have productive implications. In fact, in outdoor systems, the use of the Na genotype can lead to improved productive parameters, meat, and eggs, not just because of the ability to tolerate high temperatures, but also because these animals are more morphologically adapted and have more resistance to diseases. On the other hand, they may have fertility problems, especially the homozygous. This can lead to decreased productive parameters, especially in the number of chicks hatched per animal, with implications for financial returns.

## 5. Conclusions and Future Perspectives

Climate change leads to increasing temperatures in both tropical and temperate zones. Therefore, HS represents and will increasingly represent a worldwide challenge for poultry production. As noted in this review, the impact and severity of HS depend on the duration of exposure and temperature–humidity combination. Such an impact can be further aggravated by health, physiological stage, and general condition of the animal. Among the main effects of HS, the impact it causes on the productive performance of the animals (reduced feed intake and consequent growth) and a possible increase in mortality are the effects causing the greatest financial impact and are of greatest concern. As mentioned in this review, there are advantages and disadvantages to using the Na gene in poultry production and much of it depends on ambient temperature. The main advantages at the productive level are centered on the ability of these animals to adapt more easily to the increase in temperature, thus mitigating the impact that this increase has on productive performance. Additionally, being that more robust animals have evolved to adapt more easily to their environment, these animals are furthermore very interesting to use in industrial and alternative production systems in both temperate and tropical climates. There are several strategies to mitigate the effect of HS on animals. As mentioned in this review, the use of Na is a potential solution due to the animals’ high temperature tolerance. However, the benefit will be even greater if we combine additional strategies. Future research combining genetics with feed may be an alternative to improve the efficiency of poultry production when animals are subjected to high temperatures. This alternative may result not only in an economic benefit but also in an increase in animal welfare.

## Figures and Tables

**Table 1:** Summary of the effects of Na gene at different ages and temperatures on weight gain, feed intake, and FCR.

Age	Genotype	Temperature Ranges (°C)	Gene Effect ^1^ on Parameters			References
			Weight Gain	Feed Intake	FCR	
4–6 wk	NaNa vs. nana	32 °C	+22.8%	+12.1%	−9.7%	[58] *
4–8 wk	NaNa vs. nana	32 °C	+38.0%	+25.0%	−11.0%	[58] *
4–6 wk	Nana vs. nana	32 °C	+7.1%	+10.3%	+2.8%	[58] *
6–8 wk	Nana vs. nana	32 °C	+13.1%	+13.9%	+1%	[58] *
5–7 wk	Nana vs. nana	24–32 °C	+8.1%	+1.5%	−5.8%	[59] *
5–7 wk	NaNa vs. nana	24–32 °C	+4.5%	+2.9%	−0.7%	[59] *
0–7 wk	Nana vs. nana	18.7 °C	−0.7%	+4.5%	+5.0%	[60] **
0–7 wk	Nana vs. nana	27.8 °C	+3.1%	−4.0%	−8.5%	[60] **
0–7 wk	Nana vs. nana	31.5 °C	+4.6%	−1.9%	−7.3%	[60] **
4–6 wk	NaNa/Nana vs. nana	12–30 °C	−4.58%		=	[61] *
4–6 wk	NaNa/Nana vs. nana	32–45 °C	+14.6%		=	[61] *
0–20 wk	Nana vs. nana	26.9 °C	+12.88%	+0.4%	−5.2%	[62] **
0–20 wk	NaNa vs. nana	26.9 °C	+7.36%	+1%	−3.15%	[62] **

^1^ Gene effect of naked neck genotypes = $\frac{\left(Nana\;or\;NaNa\right)-\left(nana\right)}{nana}\times 100$ FCR = feed conversion ratio; * Trial in experimental conditions; ** Trial in commercial conditions.

**Table 2:** Results from different studies on the effect of Na gene (% live weight) on different strains at different ages and temperatures on body weight, dressing yield, feather weight, breast, and abdominal fat percentages.

Age	Genotype	Temperature Ranges (°C)	Gene Effect ^1^ on Parameters (% Live Weight)					References
			Body Weight	Dressing Yield	Feather	Breast	Abdominal Fat	
16 wk	Nana vs. nana	23–27 °C	+9.29%		−18.47%	+6.85%	−49.50%	[70] *
16 wk	NaNa vs. nana	23–27 °C	+6.82%		−43.18%	+9.54%	+59.60%	[70] *
49 d	Nana vs. nana	24–32 °C	+2.4%		−19.0%	+6.4%		[59] *
49 d	NaNa vs. nana	24–32 °C	+0.3%		−38.9%	+6.4%		[59] *
42 d	Nana vs. nana	19–26 °C	+0.5%		−10.2%		+16.7%	[71] *
42 d	Nana vs. nana	28–37 °C	+0.16%		−26.45%		−28.62%	[71] *
8 wk	Nana vs. nana	summer-autumn Max. 40–42 °C		−0.95%			=	[67] **
10 wk	Nana vs. nana	summer-autumn Max. 40–42 °C		+0.19%			+18.6%	[67] **
12 wk	Nana vs. nana	summer-autumn Max. 40–42 °C		+0.28%			+6.55%	[67] **
6 wk	Nana vs. nana	28.7–32.5 °C	+3.04%	+1.05%		+8.09%	+0.10%	[68] *
43 d	NaNa/Nana vs. nana	12–30 °C	+0.22%	+5.6%	−25.47%	+0.5%	−6.28%	[61] *
43 d	NaNa/Nana vs. nana	32–45 °C	+8.0%	+4.37%	−34.16%	+3.1%	−20.37%	[61] *
20 wk	Nana vs. nana	26.9 °C	+5.72%	+5.48%	+5.56%			[62] **
20 wk	NaNa vs. nana	26.9 °C	+3.83%	+6.96%	+7.74%			[62] **

^1^ Gene effect of naked neck genotypes = $\frac{\left(Nana\;or\;NaNa\right)-\left(nana\right)}{nana}\times 100$; * Trial in experimental conditions; ** Trial in commercial conditions.

## Data Availability

Not applicable.
